# Accessibility and availability of the Female Condom2: Healthcare provider’s perspective

**DOI:** 10.4102/curationis.v38i2.1533

**Published:** 2015-11-23

**Authors:** Seepaneng S. Phiri, Richard Rikhotso, Miriam M. Moagi, Varshika M. Bhana, Priscilla M. Jiyane

**Affiliations:** 1 Department of Nursing Science, University of Pretoria, South Africa

## Abstract

**Background:**

Despite the acceptability of the Female Condom2 (FC2) as a contraceptive method by some women, it remains inaccessible and unavailable to the majority of women because of affordability, training, distribution and marketing strategies. The FC2 affords women dual protection and the option to negotiate safe sex.

**Objective:**

This paper explores and describes the perspective of the healthcare providers regarding accessibility and availability of the FC2 as a contraceptive method in the Tshwane district.

**Method:**

The study used an explorative, descriptive, and qualitative design. Data were collected from 26 healthcare providers who were purposively selected. In-depth face-to-face interviews were conducted with these healthcare providers in the Tshwane district. Tesch’s method of open coding was used for data analysis.

**Results:**

Two main themes emerged, namely, the availability of the FC2 and the knowledge of the healthcare providers. The findings of this study indicated that the availability of the FC2 remains a challenge because of factors such as lack of affordability, inefficient procurement and lack of distribution measures. The condoms are also not available at strategic points so as to ensure accessibility. Insufficient knowledge amongst healthcare providers was described as a barrier which affects the quality of training of the service users.

**Conclusions:**

It is evident that the FC2 is not yet available in all healthcare settings, therefore strategies to safeguard accessibility and availability of the FC2 as a contraceptive method are recommended.

## Introduction

The availability and accessibility of effective methods of contraception are considered to be one of the strategies that can improve maternal health as this will reduce the rate of unplanned pregnancies (Beksinska *et al.* 2013:e151). South Africa has a high rate of unplanned pregnancies which is corroborated by the number of termination of pregnancies (TOP) performed annually. Recent statistics show that currently, out of all pregnancies, 11% are terminated yearly as compared to 13.1% in 2013 (Parnell et al. 2014:6). In Eastern and Central Europe, high rates of legally-induced TOP are reported, whereas in most of the countries in sub-Saharan Africa, TOP services are restricted by law (Lule, Singh & Chowdhury 2007:16).

The problems related to TOP globally represent 13% – 25% of maternal deaths. This could possibly decline by 25% – 35% if contraception were accessible, available and used regularly by women who want to prevent pregnancy (Opoku 2012:109). It is important to emphasise family planning before and after TOP in order to protect against sexually-transmitted infections (STIs) and HIV (Curtis, Huber & Moss-Knight 2010:44; Handlovsky, Bungay & Kolar 2012:1007). Thus the provision of a dual protection method against unintended pregnancies, STIs and HIV needs to be accessible and available to women as one of the ‘essential but underutilised’ contraception methods for improvement of maternal health (Mullan 2013:e116; World Health Organization [WHO] 2012:9). Therefore, healthcare providers need to ensure that the Female Condom2 (FC2) is both accessible and available to women.

To support women with regard to exercising autonomy in negotiation of safe sex and prevention of unintended pregnancy, the Female Condom1 (FC1) was introduced in 1993 (Guerra & Simbayi 2014:146). The initiative was welcomed by women as it was recognised as a dual protection method based on the fact that it prevents unwanted pregnancy and also STIs, including HIV (Gallo, Kilbourne-Brook & Coffey 2012:18). However, women were not satisfied with the first products because of the challenges they experienced. These challenges include, amongst others, discomfort during sex and excess lubrication, difficulty in using the device, women feeling uncomfortable inserting the condom, unavailability and affordability, partner refusal, men disliking the appearance, trust in long-term relationships and men’s condom preference (Chipfuwa et al. 2014:75). In addition, polyurethane female condoms are expensive, making it difficult for women to access and afford them (Hoffman et al. 2004:141). The dissatisfaction experienced by women with FC1 was further reported by healthcare providers in a study conducted in Tshwane (Ngunyulu et al. 2014:396). In this study, healthcare providers reported dissatisfaction with poor access to and/or unavailability of this condom.

In responding to the high price challenge of the female condom, the Joint United Nations Programme on HIV/AIDS and the condom manufacturer collaboratively invented a female condom made of latex, namely the FC2, which is cheaper than polyurethane and can be purchased and distributed by both national and international organisations (Hoffman et al. 2004:141). The FC2 was introduced in 2009 and is viewed as a better method which has awarded women negotiation power and the ability to take accountability of their health. Despite all the merits of the FC2, its accessibility and availability remains a challenge.

The intensification of the marketing of the use of male condoms as a preventive measure negatively affected the use of other family planning methods. Women started relying on male condoms as a way of prevention of pregnancy and HIV infection. The practice had challenges as women had to rely on whether a man was intending to use a condom or not. Women are still at risk, as they are unable to negotiate for safe sex (Gallo, Norris & Turner 2013:e119). In cases where men are older and financial providers, negotiation on condom use becomes more difficult, leaving women vulnerable as they do not have decision-making powers (Stern & Cooper 2014). According to Blignaut, Vergnani and Jacobs (2014:87), younger women in South African universities were also found to use condoms inconsistently, thus promoting the development of an ongoing educational programme to encourage the correct and consistent use of condoms and the necessary skills to negotiate condom use with their partners. Young women also rely on the morning after pill to correct mistakes that they make as a result of power relations that deny them opportunities to practise safe sex. Mpondo et al. (2015:8) assert that it is important to empower women with interventions which would assist them to negotiate safe sex in gender-power situations.

Mullan (2013:e116), argues that although the female condom is a good commodity to empower women to take control of their own sexuality, factors such as the procurement cost of the condom, training required for staff, marketing strategy to promote awareness and the different designs in the market make it difficult for the condom to be available in such large scales as expected. The issue of non-availability was supported by Beksinska *et al.* (2013:e146). A report by the WHO (2012) showed that the distribution of the female condom is low compared to that of the male condom and accounts for only 0.19% of global condom procurement. Ramkissoon et al. (2010:36) confirmed that since 1999, over four million female condoms were distributed per year in South Africa. However, distribution of the female condom is said to be limited to only 3.6 million at the public health facilities as compared to the 400 million male condoms which are distributed in South Africa annually.

In a study conducted in Kampala, Uganda, a sex worker indicated that poor distribution resulted in high female condom costs for buyers and this affects their income, since their services are bought at a low cost (Wanyenze et al. 2011:222). Further, Dowdy, Sweat and Holtgrave (2006:2096) indicate that it is not easy to compare the distribution of the FC2 and other products, since there is no authentic data indicating the perspectives of the society and that will remain a claim without any proof.

Health service providers’ lack of information around the female condom remains a challenge in the promotion of the FC2. In a study in New York (Mantell et al. 2011:68), health providers were unable to respond to questions such as the effectiveness of the FC2, the price range and where to purchase the condom. A healthcare provider’s response on the effectiveness of the female condom was ‘my guess is that it is slightly more effective than a male condom’ (Mantell et al. 2011:69). This signifies ignorance and lack of insight on the part of the service providers, as indicated in the study conducted by Chipfuwa et al. (2014:75) which revealed that 53% of the healthcare users surveyed did not receive proper health education, suggesting that healthcare providers are not doing enough regarding female condom awareness.

### Problem statement

Female condoms are regarded as a woman-initiated dual protection method to prevent pregnancy and protect against HIV (Beksinska et al. 2013:e151). Female condoms are recognised as an empowerment tool to assist women in negotiating for safe sex. Mantell et al. (2006:2000) supported the idea that women feel empowered and protected when they insert the FC2 themselves, without relying on the male condom which may have holes pierced in it by untrustworthy, dishonest male partners. However, despite efforts to market the condom since its inception in 1993, studies show that the condom remains unavailable to the majority of women because of factors such as affordability, training, distribution and marketing strategies.

These challenges impact on the success of developing African countries (Lee et al. 2013:1086–1087), such as South Africa, to meet Millennium Development Goals (MDGs) five and six, which reiterate the reduction of maternal and child mortality, including reduction of HIV rates. In addition, MDGs number two and three on gender equality are also affected, as the FC2 is viewed as one of the contraceptive methods allowing women to take responsibility of their own health (Ramkissoon et al. 2010:34). The distribution of the FC2 has increased worldwide to 5.1 million units. However, male condom distribution increased to 2.5 billion, creating hurdles for women in accessing the FC2 (Beksinska et al. 2013:e146). In 2010, the distribution of the FC2 increased to 11 million worldwide, but it is still inaccessible to most end-users (Mantell et al. 2015:1130).

According to a study conducted in Zimbabwe, the FC2 is expensive at retail outlets in urban areas and unavailable in rural areas, therefore, unaffordable to the population (Napieriala et al. 2008:122). In South Africa, it has been indicated that female condoms are 36 times more expensive than male condoms (Greig et al. 2008:42 and the South African government is thus reluctant to purchase the FC2.

According to the study conducted by Masvawure et al. (2014:82), the anticipated cost of the FC2 should decrease by 30% for affordability and accessibility in the prevention of HIV and other STIs. Beksinska et al. (2013:e151) alluded that inaccessibility to the FC2 may result from lack of commitment by main donors to support the programme in the prevention of HIV. Making the FC2 available and accessible to all populations should be part of the South African government’s key activities with regard to reducing and preventing the spread of HIV.

Mbarushimana and Ntaganira (2013:23) explained that theoretical availability of the FC2 is based on interventions to promote its use, but there is low utilisation amongst populations. This implies that the FC2 is not widely available in many resource-scarce countries where HIV and STIs are prevalent (Peters, Jansen & Driel 2010:124). In some developing countries, female condoms are reused because of their limited availability and affordability (Peters et al. 2010:124), posing increased risk of infection.

Hoffman et al. (2004:141) allude to the fact that widespread promotion of the FC2 faced social and political barriers, including limited advertising and promotion, higher prices, inadequate training of healthcare providers and limited distribution within the public health system. This was also supported by Mantell et al. (2011:68), who state that the healthcare providers’ knowledge about and promotion of the FC2 is limited.

Without a continuous supply of free, affordable FC2 by national and international organisations, the uptake of the FC2 is unlikely to increase. This article will explore and describe the perspective of healthcare providers whose views and recommendations were elicited with regard to accessibility and availability of the FC2.

### Aim and objective

The aim of this article is to explore and describe the perspectives of healthcare providers regarding accessibility and availability of the FC2 as a contraceptive method in Tshwane district.

### Setting

The study was conducted in the maternity, HIV and AIDs, paediatric and reproductive health clinics and hospital in Tshwane district, Gauteng province.

## Research design

The research design for this study was qualitative, exploratory, descriptive and contextual. The target population of this study were the healthcare providers in maternity, HIV and AIDs, paediatric and reproductive health units in Tshwane district, Gauteng province. Purposive sampling was utilised as this method allows for the purposeful selection of participants who have the necessary information, so that the research question and problem can be understood by the researcher (Creswell 2009:178). The healthcare providers who were fully trained and registered in their area of speciality, who had at least three years’ experience and were willing to participate were included in the study.

The study included a sample size of 26 participants and was conducted in the maternity, HIV and AIDs, paediatric and reproductive health clinics and hospital in Tshwane district, Gauteng province. Participants included allied healthcare workers (*n* = 3), clinic nurses (*n* = 3), lay counsellors (*n* = 7), lecturers (*n* = 7), nursing students (*n* = 3) and support staff (*n* = 3). The offices of the participants were used as the setting for data collection. The semi-structured in-depth face-to-face interviews were held with the healthcare providers to elicit their perspective regarding the availability and accessibility of the FC2 as a contraceptive method in Tshwane district. The following central, guiding question was used during the interviews: *What is your perspective regarding the accessibility and availability of the Female Condom2, as a contraceptive method in Tshwane district?* Probing questions on marketing, promotion, procurement and distribution strategies were also included in the interviews. The duration of the interviews varied between 30 and 45 minutes. A tape recorder was used with the consent of participants during the face-to-face interviews and the data were transcribed verbatim. Data were analysed following Tesch’s steps, as indicated by (De Vos et al. 2010:333). Through a deductive process of data analysis, two categories and five subcategories emerged. The theoretical justification of categories and subcategories was done through literature control (Polit & Beck 2008).

## Results

Two categories and five subcategories were identified and supported with literature. The results of the study are presented under the categories and subcategories listed in Table 1. This will be followed by a discussion of the findings and literature control.

**TABLE 1:** Categories and subcassstegories.

Category	Subcategory
Availability of condoms	Unavailability of female condoms at strategic points.
-	Availability of female condoms at strategic points.
-	Distribution list not communicated to all healthcare workers.
-	No procurement procedure in place.
Knowledge of the healthcare providers	Possession of insufficient knowledge regarding use.

### Availability of female condoms

The participants verbalised that women are prepared to use female condoms. The availability of the condom remains a challenge at certain clinics although it was found to be easily accessible at other clinics. Following is a discussion of the categories as provided in Table 1. In addition, Table 2 provides demographics for the participants whose views are provided.

**TABLE 2:** Profile of participants.

Participant number	Age	Gender	Years of experience	Job title	Area of employment
10	56	F	25	Operational manager	Primary healthcare
11	52	F	7	HIV and AIDS counsellor	Primary healthcare
12	57	F	8	HIV and AIDS counsellor	Primary healthcare
13	54	F	12	Health promoter	Primary healthcare
14	36	F	10	Professional nurse	Primary healthcare
15	32	F	2	Professional nurse	Primary healthcare
17	52	F	32	Professional nurse	Primary healthcare
18	58	F	31	Operational manager	Primary healthcare

#### Unavailability of female condoms at strategic points

Participants expressed that men are the ones who are catered for as their condoms are always available everywhere. One of the participants said:

‘Yes, women have the knowledge. Unfortunately female condoms are not always available like men’s condoms. They were once available in clinics and hospital during the period that nurses were being trained, now they are no longer available’ and ‘It is common for the female condom. Even now we don’t have.’ (P10)

To stress that the providers experience a severe challenge regarding the availability of the FC2, a participant uttered the words:

‘They are mostly out of stock, even now we don’t have them’ and “…they are scarce.”’ (P14)

Another participant said:

‘Sometimes we stay without stock because they don’t have transport to use to collect them.’ (P12)

Another participant mentioned:

‘Do you see I have male condoms on my table? I usually have both male and female condoms but now the female condoms are out of stock.’ (P11)

Cost was identified as a factor contributing to the unavailability of the FC2. One participant supported the statement by indicating that every time they place an FC2 order, they are told that it will be too expensive as compared to male condoms. The use of the FC2 raised hopes for the users. One participant indicated that the use of the device saves women from STIs and pregnancy. The unavailability of the items seemed to have shattered those hopes. Distribution was pointed out by one participants as being a challenge affecting availability of the FC2:

‘It’s too much hassles [*sic*].’ (P12)

Another participant echoed:

‘We have the problem in obtaining them. There is always the stock out which is always beyond our control. We get them when they are available, but in most cases we do not have stock. That is why I cannot say more about it, we get it or not. It is very scarce.’ (P10)

The healthcare providers also expressed frustration in terms of their role concerning the promotion of the condom, as it is usually unavailable. This was expressed as follows:

‘We are urging women to use the male condom since it is easily obtainable as compared to female condoms. They must just improve the availability because it makes us liars, we tell people about them and when they come they are out of stock.’ (P10)

One participant linked the issue of unavailability of the condom to the healthcare providers’ frustrations resulting from inadequate practice of the skill of condom insertion:

‘We are often not sure how to teach women as we also do not have a chance to provide the condom more often. Its unavailability makes you forget the skill easily.’ (P14)

The same sentiments were shared by another participant:

‘I have been trained. I was so excited to learn about the FC2, I wanted to promote it even with friends at home. However the hospital did not order more after the boxes that we got fr om training were finished. You have to contact the Department of Health time and again.’ (P14)

#### Availability of female condoms at strategic points

Some participants expressed their satisfaction with regard to the availability of female condoms at their centres. They also indicated that their clients were collecting them regularly. This was expressed as follows:

‘They are available we have them in stock right now.’ (P15)

Another participant said:

‘However, in certain clinics the FC2 is available and accessible’ and ‘They also indicated that the women who use the FC2 often ‘boast about how good they are and use them occasionally.’ (P11)

Another participant mentioned:

‘Yes they are available, the health promoters does deliver them. Do you see I have an empty box – it’s just finished. The female condoms have their customers who come to the clinic special for them. Some will even send me from home to bring for them. They love them.’ (P11)

The participants also expressed concern about the female condoms being available only at the clinics and hospitals. It was expressed as follows:

‘They must think broad with female condom; at the moment we only get them from the clinic only. School children need to access it as easy as the male condoms. They must put them at the shebeens and shops because at night where will they find them as the clinic will be closed.’ (P12)

This participant highlighted the need to have the FC2 available in other areas in addition to clinics, thus improving accessibility to all consumers.

#### Distribution of female condoms to healthcare providers

Participants expressed that the distribution list is often not communicated to all healthcare providers, which makes it difficult to issue and provide the female condoms to clients:

‘I think the service provider needs to improve the distribution strategy. The improvement of the FC2 distribution can be done by distributing the female condoms like male condoms; with the male condoms, the health promoters identify the sites for distribution then the truck will come and deliver to the service points, I mean the shebeens and the shops.’ (P10)

Another participant said:

‘They must balance the equation by delivering the same number of male to the same number of female condoms.’ (P14)

Participants suggested that the FC2 be made available at different sites and service points so as to make them easily available to the public.

#### No procurement procedures in place

Participants were worried that they do not have enough female condom stock and that they had challenges obtaining sufficient stock when they ordered. This was expressed as follows:

‘Sometimes we order and receive less number and even from that number we have to still share with other facilities. The stock is never adequate.’ (P10)

One participant echoed:

‘Yes, we don’t receive the same amount of female condom as male condoms and every time I ask for the reason why, they tell us that the female condoms are expensive as compared to the male condoms. Why we are even told that it’s expensive when we want the order?’ (P11)

Another participant indicated:

‘If we could just get enough stock, I think we’ll be able to get people confident about it. First of all, we don’t procure at the clinic level; the district office is the one that ensures that the procurement is complete. All we do we go to them and order.’ (P14)

It is evident in the quotes provided that female condoms were not readily available, as they were known to be expensive therefore could not be provided at different facilities.

#### Knowledge regarding the use of female condoms

Participants related how they lacked the necessary information regarding the use of the female condom and therefore were sometimes giving insufficient information regarding its use. Possession of insufficient knowledge of the female condom emerged as a subcategory.

#### Possession of insufficient knowledge regarding use

Insufficient knowledge was identified as a challenge for healthcare providers even though they were expected to educate their clients on how they are used. They expressed their feelings as follows:

‘Yes, but I never demonstrated on how to use them. [*Why?]* Because I don’t know how to use them so I just tell them that “they said you must insert it on [*sic*] four hrs before you have sex”. I think it is very much important because if I don’t have enough information I won’t feel confident on issuing them.’ (P13)

And:

‘I usually give them the leaflet to read the instruction on how to use them. [**Do you think the leaflet is user friendly?*]* Firstly, we must be educated as healthcare workers regarding the use of female condoms. They must please train us on the use of female condom; they shouldn’t just assume that we know them. If we have enough info, we’ll gladly issue and educate the clients more.’ (P15)

Another participant said:

‘We give the little information that we have. Yes we need a lot of training on it. Daily in-service training to staff until we are sure of the facts. Then we can educate the clients.’ (P17)

Another participant mentioned:

‘What can be done is continuous training; audit them to see how competent they are in using them. We need a doll to demonstrate on how to use like we do with the male condom with a dildo; that way we’ll know that they know the procedure of insertion.’ (P18)

Participants felt they lacked information regarding the use of the FC2 and were thus unable to educate women appropriately on how to use it.

## Ethical considerations

Permission to conduct the study was obtained from the following institutions: The University of Pretoria’s Faculty of Health Sciences Research Ethics Committee (Ref No. 198/2012), the Gauteng Province Department of Health, the unit managers of different institutions and a written informed consent from the participants themselves. The following principles were adhered to, in order to ensure that the participants were protected: the principle of respect and human dignity; principle of justice and informed consent; principle of non-maleficence and beneficence; and the principle of veracity and fidelity (Pera & van Tonder 2011).

The participants were reassured that all the information they gave would not be used against them in any way and that their participation was voluntary, as they had the right not to participate in the study if they so wished. Participants were given full information about the research project. Their right to make informed decisions was also covered through the contents of the participant leaflet and the consent to participate in the study. These participants were treated fairly throughout the study and their privacy was maintained through the use of pseudo-names. All the information gathered from the study was used for the purposes of the research only and was kept locked in the cabinet accessed by the research team only.

## Trustworthiness

Credibility, dependability, confirmability and transferability were adhered to in order to ensure trustworthiness. To ensure credibility, the appropriate methods of participant selection and data management were used, which included prolonged engagement, persistent observation, triangulation, peer debriefing and member checks. Dependability was achieved by ensuring that the collected data reflected the emotions, opinions and views of participants (Lincoln & Guba 1985). The study reflected the conditions of inquiry and the participants’ voices – not the researcher’s perspectives, motivations and biases; hence confirmability was achieved (Polit & Beck 2008). To achieve transferability, the researchers ensured that the results of the study reflect the perspectives of healthcare providers regarding the availability and accessibility of the FC2 as a contraceptive method which are likely to be transferrable to all healthcare providers in Tshwane district.

## Discussions

This study confirmed that accessibility and availability are factors which influence female condom use. Healthcare providers identified poor distribution and lack of female condom stock in the clinic as being challenges. To a certain extent, the unavailability of the FC2 discouraged healthcare providers from promoting its use. One of the reasons they mentioned was that when women come to collect additional female condoms, in most instances the clinics were out of stock as the supply quantities are less than required to service consumer demands. The healthcare provider verbalised this perception of clients seeing them as ‘liars’ because of the unavailability of the FC2 and were thus reluctant to promote its use. These factors existed in this study even though women were both knowledgeable about and willing to use the FC2. The enthusiasm shown by women with regard to use of the FC2 was reported by Weeks et al. (2010:303), who found that women were willing to encourage their partners to support the use of the female condom once they overcame the initial challenges of its usage. Furthermore, women who have positive experiences and speak positively about the female condom have been found to positively influence other female condom users (Weeks et al. 2010:303). Mantell, Stein and Susser (2008:95) concur that the female condom empowers and enables women. The unavailability of the FC2 needs to be corrected so that women gain continuous access to it, thus empowering women to prevent the contraction of STIs such as HIV. On the positive side, some participants reported that certain clinics have a sustained supply; as a result the women visiting those clinics have consistent access to the FC2. The sustained availability and support provided by healthcare providers contributes to acceptability of the FC2 and encourages the use thereof (Peters et al. 2010:124).

As the FC2 was out of stock for long periods, the participants saw this as being the reason for the prevailing lack of skills. As they stay for long periods without supply, they reported that they forget how to demonstrate its insertion. This would negatively impact on women’s access to the FC2 and its use would decline. Findings in a study by Holmes et al. (2008:476 indicated an increased probability in female condom use by women who receive information about it. Holmes et al. (2008:474) found that women who were not knowledgeable about the female condom were 81% less likely to use it than women who were knowledgeable. Weeks et al. (2010:303) also found that greater female condom knowledge was associated with female condom use. The perceptions of healthcare providers confirmed that women may have decreased access to information about the FC2 and this would in turn lead to non-use as availability of the FC2 is not sustained.

Apart from availability of the FC2, unlimited accessibility of the FC2 was also emphasised in the current study. Participants of the study suggested that the female condom be distributed in a variety of settings. Similar recommendations were also made by participants in studies conducted by, Mantell et al. (2008:94), where adolescent women highlighted that having contraceptive methods available in a variety of places, such as hospitals, clinics, pharmacies, supermarkets and private organisations, as well as in vending machines in public restrooms, was important. Furthermore, it was recommended that it should be distributed through community-based distributors.

The unavailability of female condoms, especially at strategic points, was identified as a serious problem in a study conducted in New York by Mantell et al. (2011:66). Participants openly mentioned the lack of female condoms at specified points, meaning that their increased demand was ignored by the providers. The reason for the decreased supply of female condoms was perceived as a lack of commitment on the part of the donors and programme implementers, as well as governments. In addition, an appeal was made that government ensures strategies for sustainable supply of the female condoms in response to increased demand by women (Sippel 2011:5).

In this study, the healthcare providers suggested that distribution should also be done in shebeens. In support of this finding, Marumo (2011:6) asserts that public-private partnerships distribute condoms at places such as taverns and diverse private companies have been encouraged to participate and maintain condo-cans. This would empower women as the FC2 needs to be inserted prior to intercourse, meaning that one could walk around with it in place and be protected in the event that coitus occurs.

The distribution was mostly affected because of a bureaucratic management approach. In this study, participants highlighted that the FC2 stock received from distributors was usually less than the amount ordered. The reduction of quantity required based on client’s FC2 uptake leads to inaccessibility and unavailability. In a study conducted in New York by Mantell et al. (2011:66), participants openly mentioned the lack of female condoms at specified points, meaning that their increased demand is ignored by the providers. Enough budget should be available so that the government secures strategies to ensure sustainable investment in the female condoms so as to give women the ability to make decisions regarding their protection from STIs and unplanned pregnancies (Sippel 2011:57).

It is concluded that, irrespective of the government’s plan to have the FC2 as available as the male condom, budget constraints and communication breakdown exist between managers and healthcare providers. These in turn have a negative effect on the accessibility and availability of the FC2.

However, health service providers experience challenges, just as the consumers do. Lack of and failure to communicate procurement procedures in South African healthcare organisations frustrate healthcare providers. Proper processes for acquiring items are a need as raised by the participants of the study. In support of the processes for acquiring the FC2, Marumo (2011:6) reported that the demand for female condoms in South Africa is growing and there are plans to expand access by ensuring that provinces are supported and that the FC2 is made available through public, private and non-governmental organisation outlets.

A procurement procedure, starting from product specification to item acceptance criteria, is practised by the health service providers in the Centre of Health and Gender Equity, Washington, DC (Florida Health 2013); this may be useful to other counties, South Africa inclusive. The WHO (2007) indicates the need for attending to product specifications before any supplies can be ordered, which may be an eye opener for South Africa when handling procurement of FC2s.

Participants mentioned that the FC2 was not readily available because they were told it was expensive compared to male condoms. In a study conducted in Brazil and South Africa by Dowdy et al. (2006:2096), it was argued that the FC2 is expensive; however, if it is distributed in large enough volumes, it could be manufactured at a lower cost. Not enough stock was usually present at the different health centres, creating problems with regard to availability. In 2009, the Minister of Health of Uganda, as cited by Wanyenze et al. (2011:222), asserted that the introduction of the FC2 may lead condom users to decide to use male condoms, since the FC2 is more expensive than male condoms.

Insufficient knowledge regarding the use of the FC2 was cited as a challenge by participants, who indicated their frustration with regard to how to guide clients to use them. Some clients were given pamphlets to read for themselves, meaning that participants were not sure if the clients understood the instructions. According to Ngunyulu et al. (2014:406), healthcare providers need to be empowered with knowledge and skills regarding how to use female condoms. Knowledge and support provided by healthcare providers contribute to acceptability of the female condom, which in turn contributes to accessibility, as positive experiences through support and knowledge encourage use of the female condom (Peters et al. 2010:121). Some health providers indicated that they lacked pelvic models to demonstrate condom use and also lacked knowledge and support to offer female condoms. Startlingly, participants indicated that they had an insufficient knowledge about the female condom.

The lack of information and support can contribute to non-use of the female condom, whereas information and support can encourage a positive experience. Further, women who have positive experiences and speak positively about the female condom have been found to positively influence other female condom users (Weeks et al. 2010). Handlovsky et al. (2012:1013) found that women relied on the media and clinic-based doctors and nurses for support with regard to condom use. This illustrates the importance of a positive community environment for the promotion and use of the female condom.

## Recommendations

This study’s findings suggest that the FC2 is accepted and acceptable to female clients. Therefore, the recommendation is that the FC2 should be made available to female clients through distribution to strategic points. Managers need to ensure that the FC2 orders are not altered as the healthcare practitioners at the operational level know the clients’ demands and FC2 uptake better than they. Finance to procure enough supply should be made available.

Counselling and counsellors are needed to help clients realise the contraceptive and disease protection potential of the female condom. Healthcare providers are sources of information and support to women who are using or are interested in using the female condom. It is therefore recommended that, in addition to having sufficient female condom stock and continuous replacement thereof, the necessary equipment required for demonstration of female condom use, such as mannequin pieces and counselling, are necessary to ensure correct and ongoing use of the female condom.

Lack of sufficient exposure of the female condom by experts, such as HIV experts, may negatively influence acceptability of the female condom by policy makers and therefore negatively influence the distribution. Healthcare professionals and policy makers will promote that which is promoted by experts through literature and conferences. This means that if little exposure is given to female condom in literature, as was found by Peters et al. (2010:121), then it is likely to be seen as less efficient than other methods which are given more exposure, such as male circumcision. This study therefore recommends that further studies on female condom use be conducted and presented at conferences. Furthermore, studies with a strong methodological foundation should be conducted in order to influence policy development and healthcare practices.

## Limitations of the study

The study was conducted in the clinics and hospitals in Tshwane district, Gauteng province, therefore the results cannot be generalised regarding accessibility and availability of the FC2 in other provinces.

## Conclusion

The study concluded that the FC2 was not readily available at the different clinics and hospital, therefore clients were not always encouraged to use them because of their unavailability. Healthcare providers raised concerns about poor distribution and procurement of the FC2 in the clinics. Educating and training clients on how to use the FC2 was a challenge to healthcare providers because they lacked the relevant knowledge regarding its use.
